# Biodiversity Loss following the Introduction of Exotic Competitors: Does Intraguild Predation Explain the Decline of Native Lady Beetles?

**DOI:** 10.1371/journal.pone.0084448

**Published:** 2013-12-27

**Authors:** Chelsea A. Smith, Mary M. Gardiner

**Affiliations:** Department of Entomology, The Ohio State University, Ohio Agricultural Research and Development Center, Wooster, Ohio, United States of America; La Trobe University, Australia

## Abstract

Exotic species are widely accepted as a leading cause of biodiversity decline. Lady beetles (Coccinellidae) provide an important model to study how competitor introductions impact native communities since several native coccinellids have experienced declines that coincide with the establishment and spread of exotic coccinellids. This study tested the central hypothesis that intraguild predation by exotic species has caused these declines. Using sentinel egg experiments, we quantified the extent of predation on previously-common (*Hippodamia convergens*) and common (*Coleomegilla maculata*) native coccinellid eggs versus exotic coccinellid (*Harmonia axyridis*) eggs in three habitats: semi-natural grassland, alfalfa, and soybean. Following the experiments quantifying egg predation, we used video surveillance to determine the composition of the predator community attacking the eggs. The extent of predation varied across habitats, and egg species. Native coccinellids often sustained greater egg predation than *H. axyridis*. We found no evidence that exotic coccinellids consumed coccinellid eggs in the field. Harvestmen and slugs were responsible for the greatest proportion of attacks. This research challenges the widely-accepted hypothesis that intraguild predation by exotic competitors explains the loss of native coccinellids. Although exotic coccinellids may not be a direct competitor, reduced egg predation could indirectly confer a competitive advantage to these species. A lower proportion of *H. axyridis* eggs removed by predators may have aided its expansion and population increase and could indirectly affect native species via exploitative or apparent competition. These results do not support the intraguild predation hypothesis for native coccinellid decline, but do bring to light the existence of complex interactions between coccinellids and the guild of generalist predators in coccinellid foraging habitats.

## Introduction

Declines of many native species have occurred simultaneously with the introduction and spread of non-native organisms, a pattern that implicates competition with exotics as a contributing factor [1], [2], [3], [4]. Often, this is based primarily on correlative evidence and lacks mechanistic understanding [5]. The influence of exotic generalist predators on native population decline is particularly difficult to elucidate, as both direct and indirect competitive interactions occur [6], [7], [8], [9]. For example, the invasion of two wasp species into New Zealand’s southern beech forests has directly and indirectly negatively affected native fauna. Following their establishment, populations of invertebrates preyed on by the wasps have declined. Additionally, the kaka, a native parrot that forages on honeydew produced by a native scale has abandoned the invaded area as up to 95% of available honeydew resources are claimed by the wasps [7].

Coccinellids (Coleoptera: Coccinellidae) are an excellent model to study these direct and indirect interactions between native and exotic competitors; they compete for resources and also interact with each other though intraguild predation (IGP) [9], [10], [11], [12], [13], [14]. Coinciding with the establishment of exotic coccinellids, several multi-year surveys have documented population declines of native coccinellid species including *Coccinella novemnotata*, *Coccinella transversoguttata*, *Adalia bipunctata*, *Hippodamia tredecimpunctata*, and *Hippodamia convergens* [15], [16], [17], [18], [19], [20], [21]. Among the most recently detected declines is that of the convergent lady beetle, *H. convergens*, within the Midwest United States [22], [23], [24], [25]. These declines have led to several hypotheses which try to explain the loss of native coccinellids following exotic introduction, including interference competition via IGP [26], [27], exploitative competition for shared prey [20], [28], and apparent competition via a shared parasitoid [29]. The majority of research examining native coccinellid decline has focused on interference competition.

Laboratory studies have documented that exotic coccinellids feed on eggs and larvae of coccinellids and other predatory insects [27], [30], [31], [32], [33], [34]. Field studies documenting the decline of native coccinellids following the introduction of exotic species are limited and the conclusions reached are often conflicted regarding the degree of IGP that occurs between species. For example, Hoogendoorn and Heimpel [35] found that in field cages, the presence of exotic *Harmonia axyridis* larvae did not reduce the survival or weight gain of native *Coleomegilla maculata* larvae. Conversely, Gardiner et al. [24] reported that *C. maculata* incurs significant egg predation in soybean fields; however, the contribution of exotic coccinellids to this mortality was not measured.

Thus, a determination of which animals consume coccinellid eggs and quantification of the incidence of egg predation for different coccinellid species in varied habitats is necessary to understand of the contribution of egg predation to native coccinellid decline. To address this, we compared the extent of egg predation experienced by three coccinellid species: a native experiencing population decline (*H. convergens*), a common native (*C. maculata*), and a common exotic (*H. axyridis*) species. The inclusion of a common native species provided an opportunity to compare egg loss of both a common native and exotic against the declining native species. This study examined levels of egg predation occurring across coccinellid foraging habitats and determined the guild of predators responsible. If egg predation has contributed to native coccinellid decline we expected consumption of declining native coccinellid eggs to exceed that of common native or exotic species. We tested the predictions that 1) exotic coccinellids are the dominant coccinellid egg predator, 2) declining native species incur greater egg predation than common species, and 3) less predation of native eggs will occur in semi-natural grasslands relative to croplands. Previous studies have found native coccinellids to be abundant in semi-natural grassland habitats as well as landscapes comprising of higher proportions of semi-natural grassland habitat implying that they may be reproductively successful there [22], [36].

## Materials and Methods

### Ethics statement

Specific permits were not required for field sites and this study did not involve protected or endangered species. Permission to gather data from fields was obtained from each grower and private landowner.

### Study sites

Data were collected within nine counties in 2009 and 2010 throughout Ohio, USA: Delaware (2010 only), Fayette, Huron, Knox, Marion (2009 only), Perry, Putnam, Shelby, and Wayne (Figure S1). Within each county one semi-natural grassland, alfalfa, and soybean site was selected; each was separated by a minimum of four km. Alfalfa and soybean fields were managed by grower-collaborators. Grasslands were owned privately and contained cool and warm season grasses, native forbs, and agricultural weeds. Four plots were established per site where data were collected; all plots were a minimum of 30 m from any field edge (Figure S2).

### Rearing of coccinellid species

*Coleomegilla maculata* and *H. axyridis* adults were collected from overwintering aggregations in Ohio. *Hippodamia convergens* adults were ordered from a commercial supplier, which collects from overwintering aggregations in California (Rincon-Vitova, Ventura, CA, USA). Adult females were placed into individual plastic vials (8.9 cm deep; 4.5 cm diameter) lined with white paper to act as an egg-laying substrate as in Gardiner et al. [24]. All beetles were provided with water, honey, and aphids. Eggs deposited onto the paper substrate were counted and stored in a –80°C freezer until experiments were conducted.

### Measuring predation of frozen egg masses

Previous research by Gardiner et al. [24] found no preference among predators for live versus previously-frozen coccinellid eggs. To further determine that previously frozen eggs could be used for field experiments, the consumption of previously-frozen and live egg masses was compared for additional predator taxa (Stylommatophora (slugs), Opiliones, Acrididae, and Tettigoniidae). Predators collected from the field (Wooster, OH, USA) were starved for 12 hours, and placed into individual containers (8 cm deep; 11 cm diameter). Each predator was provided a previously-frozen and fresh egg mass of similar size and species (*H. axyridis* or *H. convergens)*. The fresh egg masses were no older than three days. All bioassays were conducted at room temperature, and took place between May 22, 2011 and August 14, 2011. The slugs were moistened with water every 60 minutes to prevent desiccation. The number of eggs remaining within each container was determined every 30 minutes for four hours, and thereafter at 24 and 48 hours.

### Egg predation experimental procedure

To quantify egg predation in the three focal habitats, egg loss in Open (predator accessible) and Exclusion (cage used to exclude predators) treatments were compared (Figure S2A). The Exclusion treatment consisted of an egg mass enclosed in a 22 mm diameter mesh cage. This measured background egg loss due to desiccation or dislodgement to ensure that there was not an overestimate of predation events. The Open treatment consisted of an un-caged egg mass. To begin each experiment, egg masses deposited onto 1.5 cm^2^ paper squares were counted and attached to vegetation 0.2 – 0.3 m from the soil surface. Two egg masses from each species were present in each plot; one was assigned to the Open treatment and the other to the Exclusion treatment. The number of eggs within a mass ranged from 2 to 54 with a median of 8. The high variability could not be avoided due to the large number of egg masses required for the experiments. All treatments remained in the field for 48 hours, after which the egg masses were collected and the remaining eggs counted. The proportion of eggs remaining was calculated for each egg mass. The presence of predator frass or a hole in the cage mesh indicated that predators were able to gain access to 40 Exclusion cages (across 2009 and 2010). Data from infiltrated cages were excluded from analysis. For each experiment, the proportion of eggs remaining in each treatment was averaged by site for each coccinellid species.

In 2009, one egg predation experiment was conducted during the week of July 20. Predation on *C. maculata* eggs was measured within all 24 field sites (eight of each focal habitat) and predation on *H. axyridis* eggs was measured within nine sites (one of each focal habitat within Shelby, Wayne, and Perry Co., OH). In 2010, experiments were conducted during the weeks of June 8 and July 26. The June 8 experiments were carried out within the grassland and alfalfa habitats. Predation in soybean was not examined since plants had recently or not yet emerged from the ground within our sites. Egg masses of *H. axyridis* and *H. convergens* were deployed in all grassland and alfalfa sites and *C. maculata* in four replicates of each habitat (grassland and alfalfa sites in Knox, Perry, Putnam, and Shelby Co., OH). During the July 26 experiments, predation was measured in all three focal habitats. Eggs of *H. axyridis* and *H. convergens* were deployed in all 24 sites, and *C. maculata* egg masses in all but one site (alfalfa, Huron Co., OH).

### Video surveillance of egg predation events

We surveyed the arthropods responsible for egg predation using surveillance systems modified from a design by M. Grieshop [37], [38]. Each system consisted of a 4-channel digital video recorder (DVR: model QH25DVR, QSee, Anaheim, CA, USA) and four weather resistant surveillance cameras with infrared light for night vision (model QD28414C4 QSee, Anaheim, CA, USA). Systems were powered with a deep-cycle boat battery (SLI24MDC Xtreme Deep Cycle Marine and Boat, Hartland, WI, USA). Cameras were fitted with aluminum adaptors (Wayne Machine Shop, Wooster, OH, USA) allowing for the attachment of a 10x magnification lens (Figure S3).

Within each site, two DVR systems were placed in the field (n = 8 cameras). Two egg masses of *H. axyridis* and *H. convergens* were observed (n = 4 observations per egg mass species per site). Egg masses were attached to vegetation which was secured to a corrugated plastic stand (5×5 cm) that was anchored to the ground with 12-gauge wire to prevent movement of egg mass (Figure S3). Each camera was fastened to metal t-posts at a height of 0.2 – 0.3 m under a rain guard of white corrugated plastic (30×17 cm) to record activity at each egg mass. The battery powered the system for approximately 24 h and the video was downloaded from the DVR onto a portable hard drive. A total of 128 egg masses were observed with the cameras (66 *H. convergens* and 62 *H. axyridis*); 63 in grassland, 37 in alfalfa, and 28 in soybean from June 18^th^ – August 13^th^, 2010. From this video, we determined which animals damaged or consumed the coccinellid egg masses. Only the first attack on each egg mass is reported herein.

### Coccinellid activity density and relative abundance during egg predation experiments

The activity density and relative abundance of coccinellids present within each site was determined using yellow sticky card traps and sweep sampling during each of the three egg predation experiments. These surveys were conducted to determine if native and/or exotic coccinellids were in the field at the time of the experiments. Unbaited yellow sticky card traps (23×28 cm unfolded) (Pherocon AM, Trécé, Inc. Adair, OK, USA) were attached to step-in fence posts and placed at a height of 0.5 m at each experimental plot (Figure S2C). One seven day catch was collected per plot. Yellow sticky card traps sample the “activity density” of a population since they collect arthropods moving through the site. Four 20-sweep samples were also collected from the fields (one per plot) using a 15” diameter net (Figure S2B). All species counts were averaged across plots for each site.

### Aphid Abundance

In 2010 all aphids (Aphidoidea) were counted from the sweep samples and averaged across plots for each site.

### Data analysis

A mixed effects repeated measures analysis of variance model (ANOVA) was used to determine if predators had a preference for previously frozen or fresh egg masses. Separate analyses were conducted for each predator (Stylommatophora, Opiliones, Acrididae, and Tettigoniidae). The response variable in these models was the proportion of eggs remaining, which was arcsine(√*x*) transformed prior to analysis. Fixed factors were Treatment (fresh or frozen eggs) and Time (0.5, 1, 1.5, 2, 2.5, 3, 3.5, 4, 24, or 48 h); the interaction term Treatment*Time was also included. Data were analyzed using the PROC MIXED procedure in SAS [39].

To determine if egg predation varied among coccinellid species or across habitats a mixed effects ANOVA (PROC MIXED) [39] was conducted for each of the three experiments (June 20, 2009, June 8, 2010, and June 26, 2010) with fixed effects: Treatment (Exclusion and Open), Habitat, and Egg Species. All possible interaction terms among the fixed effects were also included in the models. To meet the assumptions of ANOVA, the response variable, mean proportion of eggs remaining, was arcsine(√*x*) transformed prior to analysis. Differences in least squares means were used to compare all variable combinations.

To determine if relative abundance of native and exotic coccinellids varied across habitats during egg experiments a generalized linear model with a negative binomial or Poisson distribution was used depending on which distribution best fit the data (PROC GENMOD) [39]. A Poisson distribution was the best fit for the mean exotic coccinellids on yellow sticky card traps from the July 20, 2009 experiment, and mean native coccinellids in sweep samples from the June 8, 2010 experiment. The negative binomial distribution best fit the data in all other cases. The response variables for these analyses were mean exotic or native coccinellids per plot, and Habitat was the predictor variable.

To determine if the relative abundance of aphids collected via sweep samples varied across habitats a generalized linear model with a negative binomial distribution was used (PROC GENMOD) [39]. The June 8, 2010 and July 26, 2010 experiments were each analyzed separately. The response variable was mean aphids per plot, and the predictor variable was Habitat.

## Results

### Measuring predation of previously frozen egg masses

We found no difference in consumption of live versus previously-frozen eggs for any predator examined (Stylommatophora (n = 16): F_1,138_ = 0.06, *P* = 0.812; Opiliones (n = 22): F_ 1,200_ = 0.64, *P* = 0.427; Acrididae (n = 14): F_1,120_ = 0.70, *P* = 0.403; Tettigoniidae (n = 11): F_1,89_ = 0.85, *P* = 0.359). For Stylommatophora, Acrididae, and Tettigoniidae, egg predation increased over time (Stylommatophora: F_9,138_ = 13.48, *P*<0.001; Opiliones: F_9,200_ = 0.42, *P* = 0.9224; Acrididae: F_9,120_ = 3.84, *P*<0.001; Tettigoniidae: F_9,89_ = 2.04, *P* = 0.044). However, there was no significant Treatment*Time interaction for any of the predators (Stylommatophora: F_9,138_ = 0.35, *P* = 0.957; Opiliones: F_9,200_ = 0.06, *P* = 1.000; Acrididae: F_9,120_ = 0.33, *P* = 0.965; Tettigoniidae: F_9,89_ = 0.17, *P* = 0.997).

### Egg predation experiments

**Figure 1:**
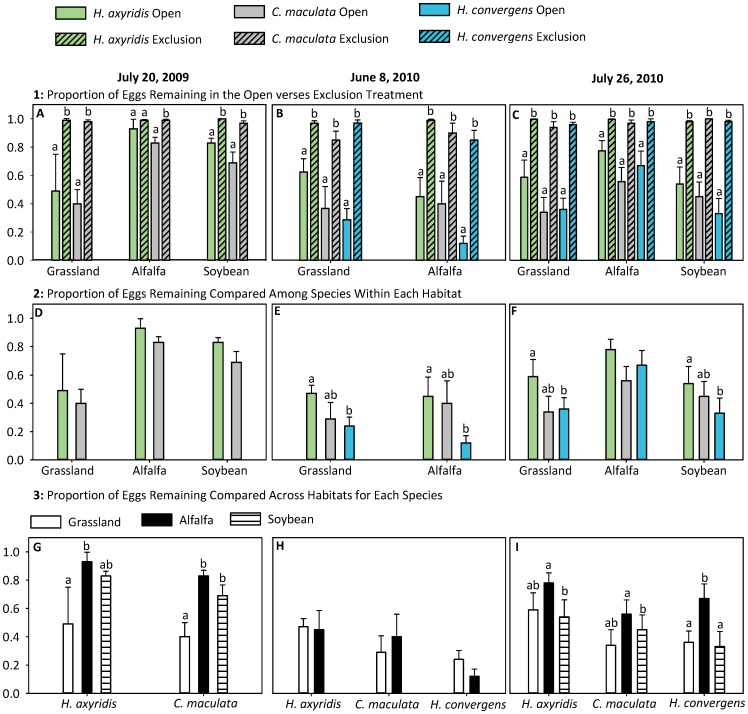
Quantification of coccinellid egg predation. The proportion of eggs remaining (Mean ± SEM) after 48 h in the field for egg predation experiments conducted the weeks of July 20, 2009, June 8, 2010 and July 26, 2010. Here we show the same data presented in three ways for clarity. Each grouping of panels focuses on different comparisons. Panels A-C show differences in the proportion of eggs remaining in Open versus Exclusion treatments. Panels D-F illustrate differences in egg predation across species within each habitat. Panels G-I show differences in egg predation within each species across habitats. Experiments were analyzed separately (*P*<0.05).

A significant interaction between Treatment (open and exclusion) and Egg Species (F_2,53_ = 4.97, *P* = 0.011) was detected for the July 20, 2009 experiments. *Coleomegilla maculata* experienced significant egg predation within all habitats, indicated by fewer eggs remaining in the Open compared to the Exclusion treatment (Figure 1a). *Harmonia axyridis* eggs were significantly reduced relative to the caged control in grassland and soybean but not in alfalfa fields (Figure 1a). The proportion of *C. maculata* and *H. axyridis* eggs remaining did not vary between habitats (Figure 1d). These species experienced similar patterns of egg predation across foraging habitats, with significantly greater egg predation occurring in grasslands than in alfalfa fields. *Coleomegilla maculata* egg masses experienced significantly greater predation in grasslands, than in soybean (*P* = 0.002) and alfalfa (*P*<0.001). There was no difference in predation of *C. maculata* egg masses within soybean and alfalfa (*P* = 0.207) (Figure 1g). *Harmonia axyridis* experienced greater egg predation in grasslands than alfalfa (*P* = 0.019) with no difference between grassland and soybean fields (*P* = 0.149). *Harmonia axyridis* egg masses in soybean and alfalfa incurred low levels of predation (82.5% and 93.3% of eggs were remaining in soybean and alfalfa respectively), with no difference in the number of eggs removed by predators (*P* = 0.275) (Figure 1g).

During the week of June 8, 2010, *H. convergens*, *C. maculata*, and *H. axyridis* incurred significant egg predation in both grassland and alfalfa habitats (F_1,68_ = 11.6, *P*<0.001) (Figure 1b). The extent of egg predation that occurred within grasslands versus alfalfa did not differ for any of the three species (F_2,68_ = 1.76, *P* = 0.190) (Figure 1h). However, there were differences in egg predation experienced among species (F_1,68_ = 5.86, *P* = 0.005). Greater predation occurred on *H. convergens* than *H. axyridis* eggs in both grassland (*P* = 0.007) and alfalfa (*P* = 0.016) (Figure 1e). There were no significant differences in the extent of predation experienced by *C. maculata* and the other two species in either of the habitats (Figure 1e).

During the week of July 26, 2010, all species sustained significant egg predation (Figure 1c). The extent of predation varied among habitats. Predation on eggs of *H. convergens* was greater in soybean (*P* = 0.002) and grassland (*P* = 0.009) than in alfalfa. There was no difference in *H. convergens* egg predation among grassland and soybean habitats (*P* = 0.625) (Figure 1i). Predation on *H. axyridis* eggs was greater in soybean relative to alfalfa (*P* = 0.048) (Figure 1i). Predation of *C. maculata* eggs in grassland was significantly higher than in alfalfa (*P* = 0.049). There was no difference in *C. maculata* egg predation among soybean and grassland (*P* = 0.409) or soybean and alfalfa (*P* = 0.210) (Figure 1i). Within foraging habitats, differences in the extent of egg predation among species were detected. In soybean fields, *H. convergens* egg masses sustained significantly greater egg predation than *H. axyridis* egg masses (*P* = 0.040) (Figure 1f). However, there was no significant difference in predation experienced by *C. maculata* and the other two focal species in soybean (Figure 1f). In grassland, significantly fewer *C. maculata* eggs remained relative to *H. axyridis* (*P* = 0.050). There was no significant difference between the amount of predation experienced by *H. convergens* and the other two focal species in grassland. In alfalfa there were no differences in predation of coccinellid eggs among any species (Figure 1f). An ANOVA table summarizing the results of the three egg predation experiments is included as a supplementary table (Table S1).

### Video observations of egg predation

**Figure 2:**
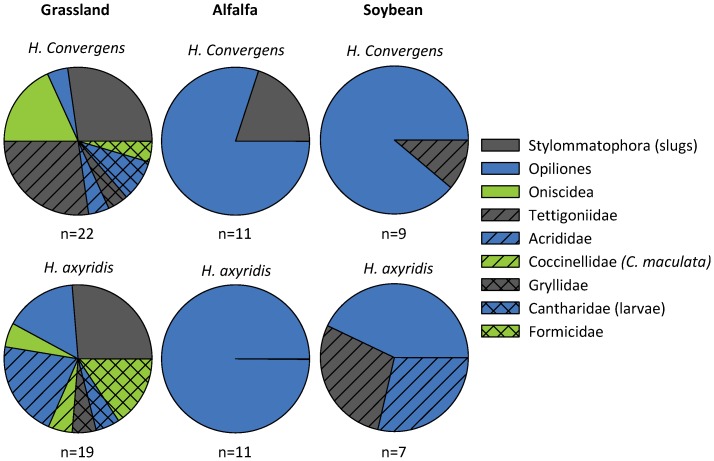
Guild of predators observed attacking coccinellid egg masses. The proportion of egg mass attacks by each predator taxa observed via video surveillance. Of 128 egg masses observed, predators attacked 41 *H. convergens* and 37 *H. axyridis* egg masses. Only the first attacked that occurred on each egg mass observed is reported here. The number of egg masses attacked in each category is reported as “n” under each pie chart.

Of 128 egg masses recorded, 41, 21, and 16 predation events were observed in grassland, alfalfa, and soybean fields respectively. We documented only one instance of a coccinellid preying on an egg mass, and this was an adult *C. maculata*, attacking *H. axyridis* eggs within a grassland habitat. Beyond coccinellids, a diverse community of organisms attacked coccinellid egg masses (Figure 2). In grassland Stylommatophora were a dominant egg predator responsible for 27.3% and 26.3% of the attacks on *H. convergens* and *H. axyridis* eggs respectively. In alfalfa Opiliones were the dominant predator, responsible for 81.1% and 100% of attacks on *H. convergens*, and *H. axyridis* egg masses respectively. In soybean Opiliones were again the dominant predator, responsible for 88.9% and 42.9% of attacks on *H. convergens* and *H. axyridis* egg masses respectively.

### Coccinellid activity density and relative abundance

**Figure 3:**
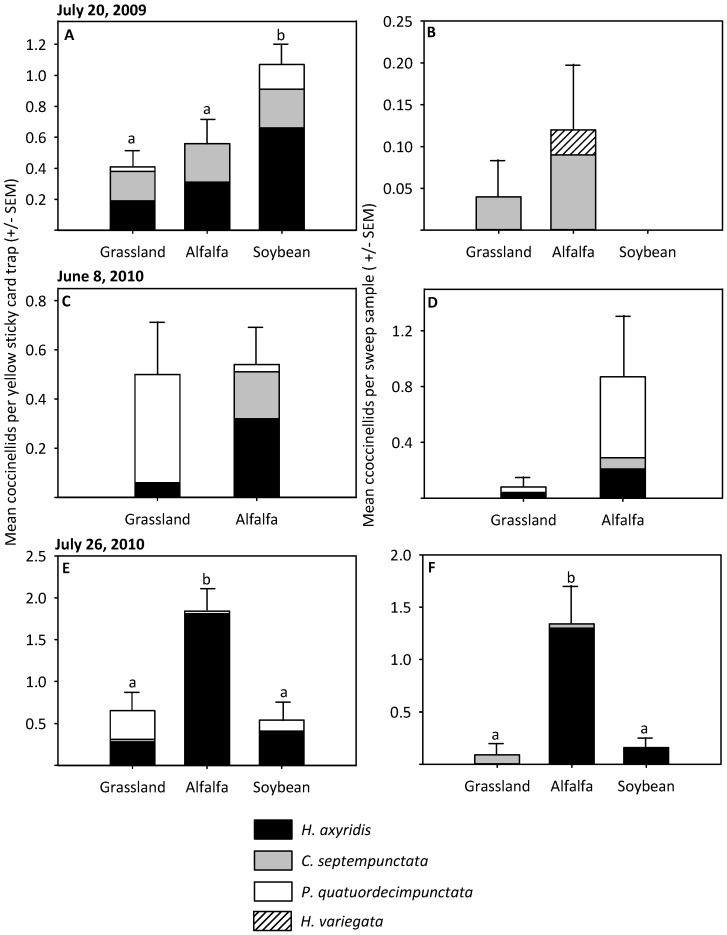
The exotic coccinellid community within field sites. The activity density and relative abundance of exotic coccinellids measured during each egg predation experiment within grassland, alfalfa and soybean habitats (*P*<0.05). A) July 20, 2009 yellow sticky card trap, B) July 20, 2009 sweep sample, C) June 8, 2010 yellow sticky card trap, D) June 8, 2010 sweep sample, E) July 26, 2010 yellow sticky card trap, F) July 26, 2010 sweep sample.

Exotic coccinellids were detected within all habitats during each egg predation experiment and *H. axyridis* was the most common species. During the week of July 20, 2009, the amount of exotic coccinellids collected from yellow sticky card traps varied across habitats (*χ* ^2^ = 10.68, d.f.  = 2, *P* = 0.005). Exotic species had a greater activity density in soybean than grasslands (*χ* ^2^ = 8.69, d.f.  = 1, *P* = 0.003) or alfalfa (*χ* ^2^ = 4.76, d.f.  = 1, *P* = 0.029) (Figure 3a). There was no difference in exotic coccinellid activity density among alfalfa and grasslands (*χ* ^2^ = 0.80, d.f.  = 1, *P* = 0.371) (Figure 3a). During the week of June 8, 2010, exotic coccinellid relative abundance sampled by yellow sticky card traps was similar in alfalfa and grasslands (Figure 3c). During the week of July 26, 2010, there was a greater activity density of exotic coccinellids in alfalfa than grasslands (*χ* ^2^ = 5.20, d.f.  = 1, *P* = 0.022) or soybean (*χ* ^2^ = 7.15, d.f.  = 1, *P* = 0.008) (Figure 3e). There was no difference in the activity density of exotic coccinellids among grasslands and soybean (*χ* ^2^ = 0.18, d.f.  = 1, *P* = 0.671) (Figure 3e).

During the weeks of July 20, 2009, and June 8, 2010, the relative abundance of exotic coccinellids sampled via sweep samples did not differ among habitats (Figure 3b, d). During the week of July 26, 2010 a greater relative abundance of exotic coccinellids was detected in alfalfa than in soybean (*χ* ^2^ = 18.58, d.f.  = 1, *P*<0.001), and grasslands (*χ* ^2^ = 18.56, d.f.  = 1, *P*<0.001) (Figure 3f).

**Figure 4:**
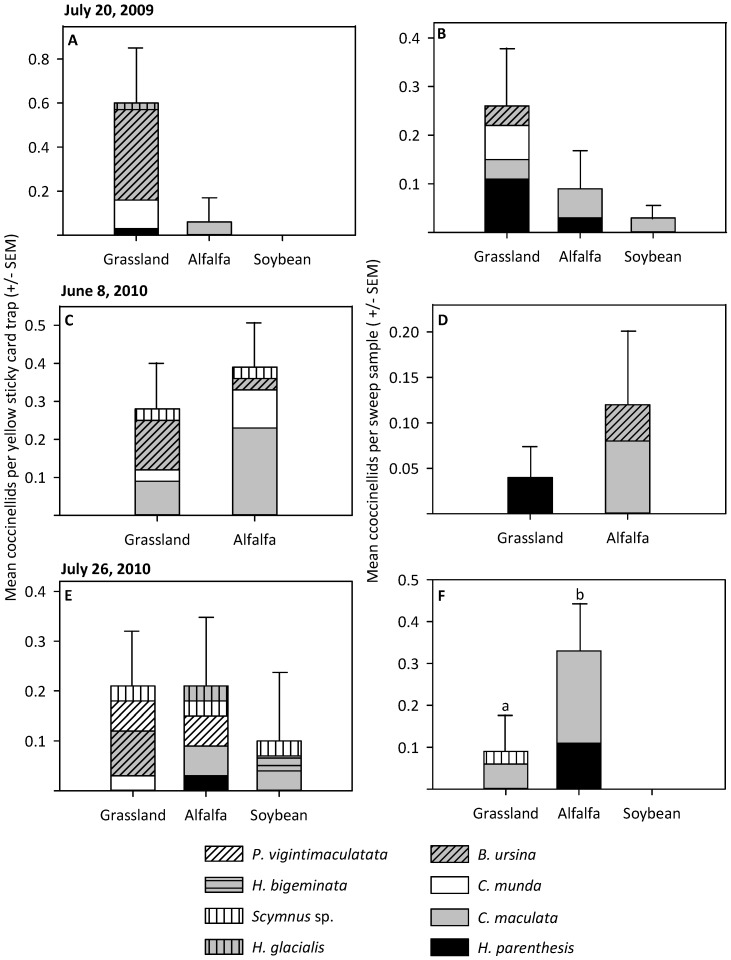
The native coccinellid community within field sites. The activity density and relative abundance of native coccinellids measured during each egg predation experiment within grassland, alfalfa and soybean habitats (*P*<0.05). A) July 20, 2009 yellow sticky card trap, B) July 20, 2009 sweep sample, C) June 8, 2010 yellow sticky card trap, D) June 8, 2010 sweep sample, E) July 26, 2010 yellow sticky card trap, F) July 26, 2010 sweep sample.

Eight native coccinellid taxa were detected throughout the 2009–2010 sampling period: *Psyllobora vigintimaculata, Hyperaspis bigeminata, Scymnus* sp., *Hippodamia glacialis, Brachiacantha ursina, Cycloneda munda, C. maculata,* and *Hippodamia parenthesis* (Figure 4). No *H. convergens* were detected during sampling in 2009 or 2010, and *C. maculata* was among the most common of the native coccinellids collected. The activity density of native coccinellids sampled via yellow sticky card traps did not vary across habitats throughout the study (Figure 4a, c, e). The relative abundance of native coccinellids collected via sweep samples also did not vary across habitats during the weeks of July 20, 2009, and June 8, 2010 (Figure 3b, d). During the week of July 26, 2010, there were fewer native coccinellids in sweep samples from grasslands compared to alfalfa (χ^2^ = 3.90, d.f.  = 1, P = 0.048), and no native coccinellids were found in soybean (Figure 4f).

### Relative abundance of aphids

During the June 8, 2010 experiment, a mean of 74.10±34.10 aphids per sweep sample were found in alfalfa whereas no aphids were recovered from the grasslands. During the July 26, 2010 experiment, alfalfa had a greater relative abundance of aphids than both grassland (*χ* ^2^ = 55.71, d.f.  = 1, *P*<0.001) and soybean (*χ* ^2^ = 58.53, d.f.  = 1, *P*<0.001), with a mean of 164.70±43.90 aphids per sweep sample from alfalfa. Sweep samples from grasslands and soybean contained 0.72±0.37 and 0.19±0.08 aphids per sweep sample respectively.

## Discussion

Worldwide, native coccinellids have declined rapidly since the introduction, establishment, and spread of exotic coccinellids [16], [17], [19], [20], [21], [40]. Research has focused primarily on direct competitive interactions among native and exotic species [27], [30], [32], [41], [42]. The propensity of exotic coccinellids, predominately *H. axyridis*, to act an intraguild predator of native coccinellid eggs and larvae has been confirmed in the laboratory. However, the extent to which declining coccinellids experience egg predation in the field and the guild of predators that contribute to this decline was not reported prior to this study.

### Role of exotic coccinellids in egg predation

Our findings did not support the prediction that exotic coccinellids are a dominant predator of declining or common native coccinellid eggs. There were detectable levels of exotic coccinellids within the focal habitats, which were observed entering the screenshots of the video experiment at times, however they were not observed consuming the egg masses. Video surveillance revealed that a diversity of organisms consume native and exotic coccinellid egg masses including harvestmen (Opiliones), ants (Formicidae), slugs, (Stylommatophora), wood lice (Oniscidae), crickets (Gryllidae), soldier beetle larvae (Cantharidae) and long and short horned grasshoppers (Tettigoniidae and Acrididae). Previous observational studies have illustrated that some of these taxa act as egg or larval predators [43], [44] but only Formicidae were commonly reported attacking coccinellid eggs elsewhere [45], [46], [47]. From our observations, Formicidae often appeared to damage eggs but not consume them, a behavior that may be triggered to protect honeydew resources [48], [49], [50]. The majority of egg predation was due to Opiliones and Stylommatophora; organisms that feed on a diversity of prey but had not previously been known to attack coccinellid eggs [51], [52], [53], [54].

### Extent of native and exotic egg predation

We found that this predator guild significantly reduced coccinellid eggs within alfalfa, soybean, and grassland habitats. Across all experiments there was only one incidence where predation on *H. axyridis* eggs did not differ from the Exclusion treatment, and this was in the alfalfa habitat. We also found variation in attack frequency among species and across habitats. If egg predation has contributed to native coccinellid decline we expected consumption of declining native coccinellid eggs to exceed that of common native or exotic species. We found partial support for this prediction. No differences in egg predation among *H. convergens* and *C. maculata* were found. However, both native species’ lost a greater proportion of eggs to predation than *H. axyridis* within some, but not all, habitats and time periods examined. The amount of predation on *H. convergens* eggs was high, with up to 88% of eggs removed within 48 hours. Additionally, up to a 33.3% difference in the proportion of *H. convergens* versus *H. axyridis* eggs removed by predators was found.

This leads to the question, why was a diverse generalist predator guild more likely to consume native versus exotic coccinellid eggs? Coccinellids are known to have species specific alkaloids present on the surface and within their eggs which may signal toxicity and deter predators [55]. Coccinellids are often reluctant to consume heterospecific eggs which can decelerate development or cause death [55], [56], [57]. Although a majority of studies investigate the effect of these alkaloids on heterospecific coccinellids, it is possible that the differences in defensive chemicals could explain why the predator guild we observed tended to consume the *H. convergens* eggs more readily than *H. axyridis* eggs. Kajita et al. [56] examined variation in quantities of alkaloids in coccinellid eggs and found that concentrations can vary significantly, both within the same species and among species.

Although exotic coccinellids are not a dominant predator of native coccinellid egg masses, reduced egg predation may confer an advantage to *H. axyridis* over competitors. Less predation on *H. axyridis* eggs could explain a higher abundance allowing them to consume more available shared resources such as aphids.

### Variation in egg predation across habitats

We also found that habitat affected egg predation risk. Across species and experiments, egg predation was either equivalent or greater within grasslands relative to the croplands studied thereby refuting our prediction that native coccinellid egg predation is reduced within grasslands. This prediction was based on the findings of several studies indicating that native coccinellids are abundant in grassland habitats relative to crop fields [58], [59], [60]. Our study illustrated that alfalfa fields offered the highest reproductive success for both native and exotic coccinellids. Eggs of all species within alfalfa fields were attacked least often in late July in both 2009 and 2010. This may have been due to greater aphid availability, as aphid counts were significantly greater in alfalfa fields in late July 2010, with 164.7 aphids per sweep sample versus fewer than one aphid per sample in the grassland and soybean fields. Extraguild prey abundance can affect intraguild predation among generalist predators [45], [61]; a pattern of decreased predation with higher extraguild prey abundance is often reported [42], [62], [63].

### Conclusions and implications

Our findings did not support the hypothesis that egg predation by exotic coccinellids has contributed to native coccinellid decline. This does not verify that exotic coccinellids cannot act as intraguild predators. Despite a lack of evidence for IGP between native and exotic coccinellids presented here, native larval coccinellid alkaloids have been detected in 121 of 590 *H. axyridis* larvae collected from lime trees in Brussels, Belgium. Native alkaloids within *H. axyridis* specimens collected indicate that native coccinellids had been consumed [63]. However, it is unknown if the level of predation was sufficient to affect the targeted native coccinellid population.

Despite the lack of direct competition found in this study, we did find that *H. convergens* sustained higher egg loss relative to *H. axyridis*. We do not have sufficient evidence to conclude that this has contributed to the decline of this species. There are no known measurements of coccinellid egg predation prior to the establishment of exotic coccinellids within the US. Thus, we do not know whether native egg predation rates have changed during the timeframe of decline. Still, it is clear that throughout these experiments, *H. axyridis* often had a reproductive advantage over native species within croplands and grasslands. Reduced egg predation of this exotic coccinellid by generalist predators may have aided its rapid population increase within its invaded range. This advantage may indirectly affect native coccinellids via mechanisms such as enhanced exploitative or apparent competition [20], [29].

## Supporting Information

Figure S1 **Site Map.** Map depicting the locations of field sites within Ohio, USA during the 2009-10 study.
(TIF)

Figure S2 **Description of experimental set up in field.** Four plots were established per site (depicted with circles 1-4). All plots were a minimum of 30 meters from any field edge. Data was collected at these plots including: Egg predation experiment with open and exclusion treatments (A), sweep sampling for coccinellid abundance (B), and yellow sticky card trap sampling for coccinellid activity density (C).
(TIF)

Figure S3 **Description of experimental set up for camera experiments**. Systems were placed randomly at two of the four plots (described in Figure S2). Systems consisted of a DVR powered by a deep cycle boat battery (B). Four cameras were connected to the DVR by a 28.3m power and video cord. Each camera was focused on a platform where a coccinellid egg mass was placed (A). All cameras were a minimum of 30 meters from any field edge.
(TIF)

Table S1 **Egg predation experiments ANOVA table.** ANOVA table for the effect of Treatment (Open or Exclusion), Species (*C. maculata*, *H. convergens*, or *H. axyridis*), and Habitat (alfalfa, soybean, or semi-natural grassland) on the proportion of coccinellid eggs remaining after 48 hours of exposure.
(PDF)
