# Supplementary figures and images for: Biodiversity Loss following the Introduction of Exotic Competitors: Does Intraguild Predation Explain the Decline of Native Lady Beetles?
Source: PLoS One. 2013 Dec 27;8(12):e84448. doi: 10.1371/journal.pone.0084448 (PMC3874011; doi:10.1371/journal.pone.0084448)

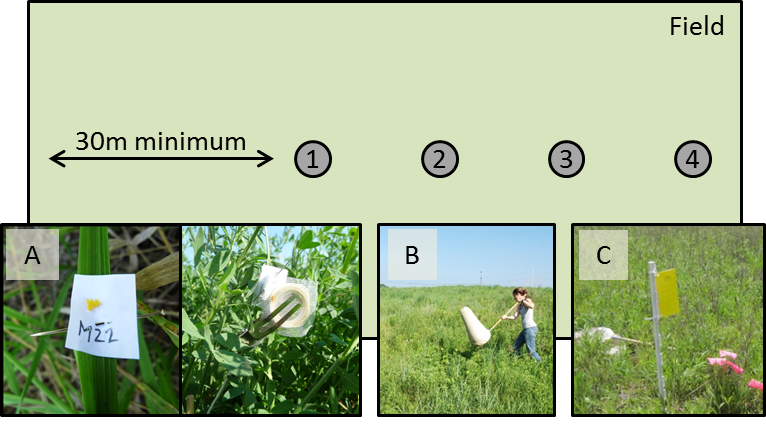

Supplement: Figure S2 — Description of experimental set up in field. Four plots were established per site (depicted with circles 1-4). All plots were a minimum of 30 meters from any field edge. Data was collected at these plots including: Egg predation experiment with open and exclusion treatments (A), sweep sampling for coccinellid abundance (B), and yellow sticky card trap sampling for coccinellid activity density (C). (TIF) [file pone.0084448.s002.tif]

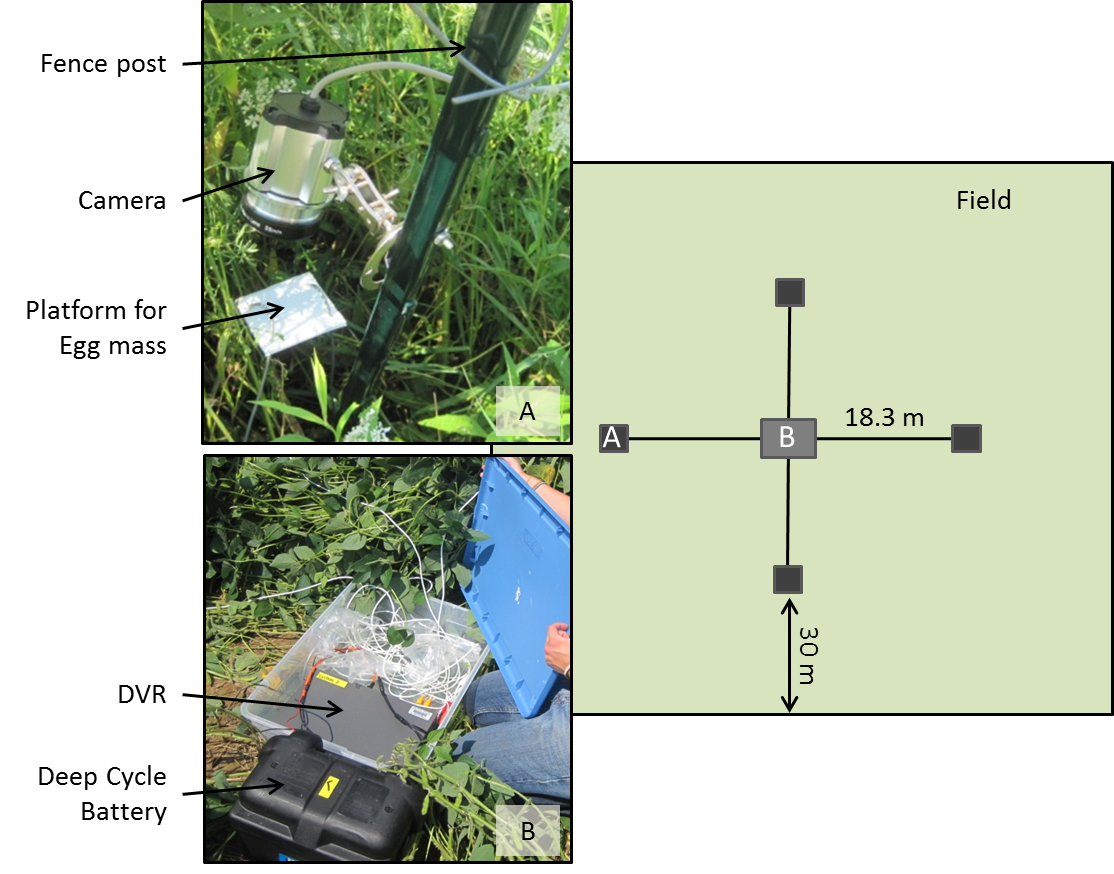

Supplement: Figure S3 — Description of experimental set up for camera experiments. Systems were placed randomly at two of the four plots (described in Figure S2). Systems consisted of a DVR powered by a deep cycle boat battery (B). Four cameras were connected to the DVR by a 28.3m power and video cord. Each camera was focused on a platform where a coccinellid egg mass was placed (A). All cameras were a minimum of 30 meters from any field edge. (TIF) [file pone.0084448.s003.tif]
