# Supplementary material for: Biodiversity Loss following the Introduction of Exotic Competitors: Does Intraguild Predation Explain the Decline of Native Lady Beetles?
Source: PLoS One. 2013 Dec 27;8(12):e84448. doi: 10.1371/journal.pone.0084448 (PMC3874011; doi:10.1371/journal.pone.0084448)
Supplement: Table S1 — Egg predation experiments ANOVA table. ANOVA table for the effect of Treatment (Open or Exclusion), Species (C. maculata, H. convergens, or H. axyridis), and Habitat (alfalfa, soybean, or semi-natural grassland) on the proportion of coccinellid eggs remaining after 48 hours of exposure. (PDF) [file pone.0084448.s004.pdf]

Year	Interaction	DF	F-value	P-value
**2009**	Treatment	1,53	48.09	**<0.0001**
	Species	1,53	2.87	0.096
	Habitat	2,53	5.36	**0.0076**
	Treatment*Species	1,53	0.92	0.3411
	Species*Habitat	2,53	0	0.9986
	Treatment*Habitat	2,53	4.97	**0.0105**
	Treatment*Habitat*Species	2,53	0.25	0.776
**2010: June**	Treatment	1,68	116.6	**<0.0001**
	Species	2,68	5.68	**0.0052**
	Habitat	1,68	1.76	0.1896
	Treatment*Species	2,68	2.38	0.1002
	Species*Habitat	2,68	1.18	0.3133
	Treatment*Habitat	1,68	0.95	0.3342
	Treatment*Habitat*Species	2,68	0.49	0.6151
**2010: July**	Treatment	1,117	213.26	**<0.0001**
	Species	2,117	3.61	**0.0302**
	Habitat	2,117	4.91	**0.0089**
	Treatment*Species	2,117	1.39	0.2543
	Species*Habitat	4,117	0.69	0.6023
	Treatment*Habitat	2,117	4.12	**0.0187**
	Treatment*Habitat*Species	4,117	0.1	0.9808
